# Dietary Nitrates, Nitrites, and Nitrosamines Intake and the Risk of Gastric Cancer: A Meta-Analysis

**DOI:** 10.3390/nu7125505

**Published:** 2015-12-01

**Authors:** Peng Song, Lei Wu, Wenxian Guan

**Affiliations:** 1 Department of General Surgery, Nanjing Drum Tower Hospital, The Affiliated Hospital of Nanjing University Medical School, Nanjing 210008, China; pengsong90@126.com; 2 Department of Laboratory Medicine, The First Affiliated Hospital of Nanjing Medical University, Nanjing 210029, China; leiwu90@gmail.com

**Keywords:** diet, nitrates, nitrites, nitrosamines, gastric cancer

## Abstract

The potential associations between dietary consumption of nitrates, nitrites, and nitrosamines and gastric cancer risk have been investigated by several studies, but yielded inconclusive results. We conducted a meta-analysis to provide a quantitative assessment of their relationships. Relevant articles were identified by a systematic literature searching of PubMed and Embase databases prior to August 2015. Random-effects models were employed to pool the relative risks. A total of 22 articles consisting of 49 studies—19 studies for nitrates, 19 studies for nitrites, and 11 studies for *N*-nitrosodimethylamine (NDMA)—were included. The summary relative risk of stomach cancer for the highest categories, compared with the lowest, was 0.80 (95% confidence interval (CI), 0.69–0.93) for dietary nitrates intake, 1.31 (95% CI, 1.13–1.52) for nitrites, and 1.34 (95% CI, 1.02–1.76) for NDMA (*p* for heterogeneity was 0.015, 0.013 and <0.001, respectively). The study type was found as the main source of heterogeneity for nitrates and nitrites. The heterogeneity for NDMA could not be eliminated completely through stratified analysis. Although significant associations were all observed in case-control studies, the cohort studies still showed a slight trend. The dose-response analysis indicated similar results as well. High nitrates intake was associated with a weak but statistically significant reduced risk of gastric cancer. Whereas increased consumption of nitrites and NDMA seemed to be risk factors for cancer. Due to the lack of uniformity for exposure assessment across studies, further prospective researches are warranted to verify these findings.

## 1. Introduction

Over recent decades, the incidence and mortality rate of gastric cancer showed a modest decline globally. In 2012, an estimated almost one million new stomach cancer cases and 700,000 deaths occurred, making it the fifth most common malignancy and the third leading cause of cancer related deaths worldwide [1]. Nearly half of the world’s new cases occurred in Eastern Asia, especially China. Geographic differences were observed in gastric cancer incidence, indicating that some modifiable factors (e.g., diet) could play a critical role in the etiology of this malignancy [2,3]. Therefore, it is an urgent demand to identify risk factors that can have a marked impact on this disease.

The typical diet in most countries contains nitrates, nitrites, and nitrosamines. Nitrates and nitrites occur naturally in fruit and vegetables, which are regarded as an important part of a healthy diet due to the powerful evidence of beneficial health effects against cancer [4,5]. In the same time, nitrates and nitrites are often used as food additives in processed meats such as ham, bacon, sausages, and hot dogs, to retard microbial spoilage, and preserve meat products recognizable appearance and flavor as well. A high consumption of processed meats is linked to an increased gastric cancer risk, and many people consider nitrates/nitrites as the main reason for that [6]. Nitrosamines are produced by chemical reactions of nitrates, nitrites and other proteins. *N*-nitrosodimethylamine (NDMA) is one of the most frequently occurring nitrosamines in our dietary foods [7,8]. NDMA is a potent carcinogen, capable of inducing malignant tumors in various animal species in a variety of tissues, including liver, lung, and stomach [9,10].

So far, numerous epidemiologic studies have been published which attempted to assess the potential risk of gastric cancer about the dietary nitrates, nitrites, and nitrosamines intake, but yielded discrepant findings. It would be of interest to evaluate, on the basis of current epidemiologic data, whether consumption of nitrates, nitrites, and NDMA had an effect on gastric carcinogenesis. To clarify their relations, we systematically reviewed all the available evidence and conducted a meta-analysis.

## 2. Methods

### 2.1. Literature Search and Selection

We searched PubMed and Embase databases through August 2015 using the following search terms: (1) nitrate AND (gastric cancer OR stomach cancer); (2) nitrite AND (gastric cancer OR stomach cancer); and (3) (nitrosamine OR *N*-nitrosodimethylamine OR NDMA) AND (gastric cancer OR stomach cancer). The manual search was supplemented by scrutinizing the reference lists from those retrieved articles to identify any relevant studies.

For inclusion, the study must meet the following criteria: (1) cohort or case-control design; (2) exposure of interest was dietary nitrates, nitrites, and NDMA intake; (3) the endpoint of interest was gastric cancer; (4) risk estimates with corresponding 95% confidence intervals (95% CIs) were provided; (5) published in English. When study populations were overlapped or duplicated in some studies, we chose the most complete and suitable research. Three authors evaluated the retrieved literature and any discrepancy was resolved through discussion.

### 2.2. Data Extraction and Quality Assessment

Two investigators using a standardized form to extract the following characteristics independently: first author’s name, publication year, population information, study location and period, sample size, follow-up years, nitrates and nitrites and NDMA intake assessment, and relative risk (RR)/hazard ratio (HR)/odds ratio (OR) with 95% CI from the most fully adjusted model for each category.

Quality of the included studies was assessed using Newcastle-Ottawa Scale (NOS) with a score ranging from 0 to 9 [11]. Each study was evaluated based on three aspects: selection, comparability, and assessment of outcome or ascertainment of exposure. Studies with score ≥ 7 were defined as being of high quality.

### 2.3. Statistical Analysis

RR was used to measure the association between the dietary nitrates, nitrites, and NDMA intake and the risk of stomach cancer. Because the absolute risk of gastric cancer was low, OR and HR approximated the RR [12]. Considering the variations within-study and between-study, a random-effects model was employed to calculate the summary RR by pooling each study risk estimate. Statistical heterogeneity among studies was assessed with the χ^2^-based Q and *I*^2^ index. If three or more studies were available for the same characteristic, subgroup analyses were conducted.

For the dose-response analysis, we used the method proposed by Orsini *et al.* to calculate the risk trend [13]. This method required the number of case and control subjects, or cases and person-years, and median level of dietary nitrates or nitrites or NDMA intake for at least three quantitative exposure categories. The median or mean consumption of each category was assigned to the corresponding dose of consumption. We assumed that open-ended category had the same amplitude as the ahead or behind category. Potential nonlinear association was assessed using restricted cubic splines with four knots at percentiles 5%, 35%, 65%, and 95% of the distribution. If linear dose-response regression with no heterogeneity was detected, we used it directly.

Meta-regression was employed to explore the possible heterogeneity, and study design, geographic area, and publication year were examined in the model. We also undertook sensitivity analysis to evaluate whether a single study could affect the overall outcome. Publication bias was assessed by funnel plot, with Egger’s regression asymmetry test and Begg’s adjusted rank correlation test, and the Duval and Tweedie “trim and fill” method was performed if bias was detected [14]. All analyses were completed using STATA version 12.0 (Stata, College Station, TX, USA). A two-sided *p* < 0.05 was considered statistically significant.

### 2.4. Disease Assessment and Dietary Assessment

As indicated above, the scope of this meta-analysis was the association between dietary nitrates/nitrites/NDMA intake and stomach cancer. The cases should be confirmed with reliable medical records such as surgical, pathology reports or linkage of authoritative tumor registries. The methods of exposure ascertainment will be extracted, which could vary considerably by the following factors: estimates from various food items and based on different food composition databases.

## 3. Results

### 3.1. Literature Search and Quality Assessment

**Figure 1:**
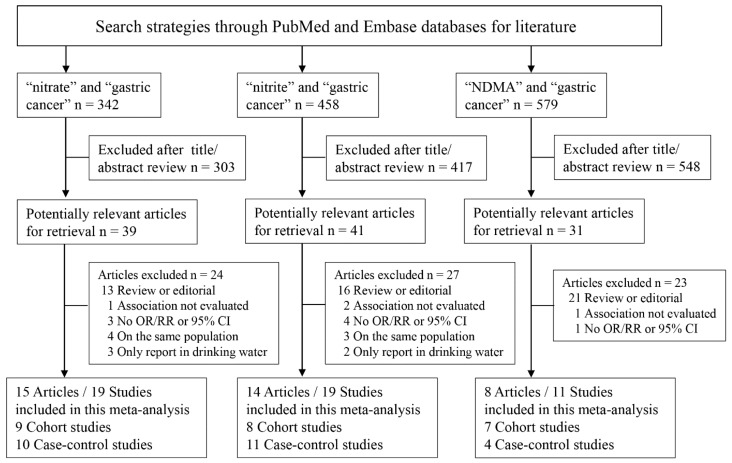
Flowchart of literature search and articles identified.

Based on the search strategy, a total of 22 articles consisting of 49 studies were included in our meta-analysis (Figure 1). Of the 22 papers, there were seven prospective cohort studies and 15 case-control studies. Among them, 15 eligible articles (19 studies) were retrieved for nitrates, 14 articles (19 studies) analyzed nitrites, and eight articles (11 studies) focused on NDMA. Table 1 and Table 2 showed the detailed characteristics of these studies. Most of the studies were carried out in North America and Europe. The publication years were from 1985 to 2013. The sample size ranged from 220 to 494,979 and the number of gastric cancer patients varied from 79 to 1016. Methods of dietary exposure differed across studies. Briefly, all included studies generally used a questionnaire to assess dietary nitrates/nitrites/NDMA intake, and that was computed by multiplying the frequency of intake of each unit of food item by the nutrient content values from food composition databases. Studies included met quality criteria of 6–9 stars (Supplemental Table S1 and Supplemental Table S2).

**Table 1:** Characteristics of prospective cohort studies in the meta-analysis.

First Author, Year, Location	Cohort Size	Follow-up (Years)	No. of Cases (Age/Definition)	Intake Assessment	Analytical Category	Definition/Nutrient Content Values	Consumption Categories	Adjusted RR (95% CI)	Adjusted Variables
Galanis, 1998 [15], Hawaii	5610 men and 6297 women	14.8	108 (NA/form Hawaii Tumor Registry, a member of the Surveillance, Epidemiology, and End Results (SEER) program of the National Cancer Institute)	short questionnarire (weekly frequency of intake of 13 foods and food groups, and the daily frequency of intake of 6 beverages)	Nitrates times/week	combined frequency of intake of dried fish, pickled vegetables and processed meats/based on previous published literature	0–3	1.0 (Referent)	Age, years of education, Japanese place of birth, gender. Analyses among men were also adjusted for cigarette smoking and alcohol intake status
							4–7	1.30 (0.80–2.00)	
							≥8	0.90 (0.50–1.40)	
Van Loon, 1998 [16], the Netherlands	1688 men and 1812 women	6.3	282 (mean: 63.0 years, SD: 4.1/exclude cases self-reported, *in situ* carcinoma, or without microscopically confirmed diagnosis)	150-item semiquantitative FFQ	Nitrates mg/day	derived from vegetables (considered loss during preparation) and drinking water/from State Institute for Quality Control of Agricultural Products solely on the intake of cured meat/from TNO Nutrition and Food Research Institute	59.8	1.0 (Referent)	Age, sex, smoking, education, coffee consumption, intake of vitamin C and beta-carotene, family history of stomach cancer, prevalence of stomach disorders, use of refrigerator or freezer
							84.7	1.25 (0.84–1.86)	
							104.4	0.74 (0.47–1.15)	
							127.3	0.92 (0.59–1.44)	
							179.8	0.90 (0.53–1.55)	
					Nitrites mg/day		0.01	1.0 (Referent)	
							0.04	1.20 (0.78–1.86)	
							0.09	1.18 (0.77–1.82)	
							0.16	0.88 (0.56–1.37)	
							0.35	1.44 (0.95–2.18)	
Knekt, 1999 [17], Finnish	9985 men and women	24	68 (15–49 years: 18 cases, 50–59 years: 28, cases, 60–99 years: 22 cases/through the nationwide Finnish Cancer Registry)	pre-formed qeustionnaire	Nitrates Quartiles	derived from vegetables (91.9%)/based on foods tables in Finland and other countries in northern Europe	Q1	1.0 (Referent)	Age, sex, municipality, smoking and energy intake
							Q2	1.01 (0.56–1.84)	
							Q3	0.52 (0.25–1.08)	
							Q4	0.56 (0.27–1.18)	
					Nitrites Quartiles	derived mainly from cured meats and sausages (94.2%)/based on foods tables in Finland and other countries in northern Europe	Q1	1.0 (Referent)	
							Q2	1.10 (0.58–2.11)	
							Q3	1.88 (1.01–3.49)	
							Q4	0.71 (0.28–1.78)	
					NDMA Quartiles	derived from smoked and salted fish (51.9%), cured meats and susages(48.1%)/based on foods tables in Finland and other countries in northern Europe	Q1	1.0 (Referent)	
							Q2	1.03 (0.55–1.95)	
							Q3	0.78 (0.39–1.56)	
							Q4	0.75 (0.37–1.51)	
Jakszyn, 2006 [8], European	153,447 men and 368,010 women	6.6	314 (mean: 59.2 years, SD: 7.48/confirmed by a panel of pathologists)	country-specific validated questionnaires	NDMA Tertiles	matched food items on the country-specific questionnaires with a food database of potential carcinogens/based on country-specific values	T1 T2 T3	1.0 (Referent)	Full cohort analysis: stratified center and age. Sex, height, weight, education level, tobacco smoking, cigarette smoking intensity, work and leisure physical activity, citrus and non-citrus fruits intake, vegetables intake, energy intake and nitrites
								0.87 (0.64–1.20)	
								0.99 (0.69–1.41)	
								Cardia	
								1.0 (Referent)	
								0.74 (0.41–1.34)	
								0.68 (0.34–1.37)	
								Non-cardia	
								1.0 (Referent)	
								1.04 (0.66–1.63)	
								1.09 (0.65–1.81)	
Larsson, 2006 [18], Sweden	61,433 women	18	156 (NA/through linkage of the study population with the national and regional Swedish Cancer registers)	67-item FFQ (before 1997) 97-item FFQ (after 1997)	NDMA μg/day	estimated by matching questionnaire food items/based on foods tables on the Swedish market	<0.041	1.0 (Referent)	Age, education, body mass index, intakes of total energy, alcohol, fruits and vegetables
							0.041–0.078	1.03 (0.61–1.77)	
							0.079–0.120	1.66 (1.00–2.75)	
							0.121–0.193	1.60 (0.93–2.76)	
							≥0.194	1.96 (1.08–3.58)	
Cross, 2011 [19], USA	295,305 men and 199,674 women	10	532 (NA/through probabilistic linkage with state cancer registries)	124-item FFQ	Nitrates μg/1000kcals	derived from processed meats/using a database of measured values from ten types of processed meats in US	24.2 66.9 112.7 174.5 298.0	Cardia	Age, education, sex, BMI, ethnicity, smoking, alcohol drinking, physical activity daily intake of fruits , vegetables, saturated fat and calories
								1.0 (Referent)	
								1.17 (0.77–1.77)	
								0.64 (0.40–1.02)	
								0.94 (0.61–1.45)	
								0.81 (0.52–1.25)	
								Non-cardia	
								1.0 (Referent)	
								0.90 (0.60–1.35)	
								0.89 (0.59–1.33)	
								0.91 (0.61–1.37)	
								1.04 (0.69–1.55)	
					Nitrites μg/1000kcals	processed meats/using a database of measured values from ten types of processed meats in US	12.1 34.6 61.4 102.9 199.2	Cardia	
								1.0 (Referent)	
								0.72 (0.47–1.11)	
								0.88 (0.58–1.32)	
								0.87 (0.58–1.31)	
								0.71 (0.47–1.08)	
								Non-cardia	
								1.0 (Referent)	
								0.77 (0.51–1.15)	
								0.79 (0.53–1.18)	
								1.04 (0.71–1.52)	
								0.93 (0.63–1.37)	
Keszei, 2013 [20], the Netherlands	120,852 men and women	16.3	663 (Women, Cardia, mean: 62.6 years, SD: 4.2; Women, Non-cardia, mean: 62.6 years, SD: 4.3; Men, Cardia, mean: 61.4 years, SD: 4.1; Men, Non-cardia, mean: 62.4 years, SD: 4.0/through linkage to the	questionnaire including 150 items on food	Nitrates Tertiles (mg/day)	derived from summing dietary intake (considered loss during preparation) and nitrate from water/based on databank of the State Institute for Quality Control of Agricultural Products	T1: women 66.4; men, 68.1 T2: women, 98.5; men 100.8 T3: women 142.7; men 146.2	Women, Cardia	Age, smoking status, years of cigarette smoking, number of cigarettes smoked per day, total energy intake, BMI, alcoholic intake, vegetable intake, fruit intake, level of education, and nonoccupational physical activity
								1.0 (Referent)	
								1.01 (0.30–3.42)	
								1.61 (0.32–8.06)	
								Women, Non-cardia	
								1.0 (Referent)	
								0.73 (0.47–1.11)	
								0.78 (0.44–1.39)	
								Men, Cardia	
								1.0 (Referent)	
								1.06 (0.68–1.65)	
								1.01 (0.57–1.77)	
								Men, Non-cardia	
								1.0 (Referent)	
								1.23 (0.90–1.68)	
								1.05 (0.70–1.59)	
			Netherlands Cancer Registry and the Nationwide Network and Registry of Histo- and Cytopathology in the Netherlands)		Nitrites Tertiles (mg/day)	processed meat/based on analyses conducted by the National Public Health Institute in 1984	T1: women, 0.02; men 0.03 T2: women, 0.08; men 0.12 T3: women, 0.20; men 0.28	Women, Cardia	Age, smoking status, years of cigarette smoking, number of cigarettes smoked per day, total energy intake, BMI, alcoholic intake, vegetable intake, fruit intake, level of education, and nonoccupational physical activity
								1.0 (Referent)	
								0.97 (0.36–2.58)	
								0.62 (0.20–1.90)	
								Women, Non-cardia	
								1.0 (Referent)	
								0.94 (0.62–1.41)	
								1.08 (0.71–1.63)	
								Men, Cardia	
								1.0 (Referent)	
								0.80 (0.51–1.27)	
								1.18 (0.75–1.86)	
								Men, Non-cardia	
								1.0 (Referent)	
								1.10 (0.80–1.50)	
								1.23 (0.89–1.70)	
					NDMA Tertiles (μg/day)	*N*-nitrosodimethylamine values in food items together with the frequency of consumption and serving sizes/*N*-nitrosodimethylamine value for food items used in the Netherlands Cohort Study	T1: women, 0.03; men 0.04 T2: women, 0.04; men 0.08 T3: women, 0.07; men 0.25	Women, Cardia	Age, smoking status, years of cigarette smoking, number of cigarettes smoked per day, total energy intake, BMI, alcoholic beverages not including beer, vegetable intake, fruit intake, level of education, and nonoccupational physical activity.
								1.0 (Referent)	
								0.97 (0.34–2.78)	
								1.02 (0.33–3.14)	
								Women, Non-cardia	
								1.0 (Referent)	
								1.37 (0.92–2.02)	
								0.90 (0.58–1.42)	
								Men, Cardia	
								1.0 (Referent)	
								1.00 (0.64–1.56)	
								0.94 (0.59–1.49)	
								Men, Non-cardia	
								1.0 (Referent)	
								1.09 (0.79–1.50)	
								1.31 (0.95–1.81)	

NDMA: *N*-nitrosodimethylamine; RR: relative risk; CI: confidence interval; BMI: body mass index; FFQ: food frequency questionnaire; SD: standard error; NA: Not Applicable.

**Table 2:** Characteristics of case-control studies in the meta-analysis.

First Author, Year, Location	No. of Cases (Age/Definition)	No. and Type of Controls	Study Period	Intake Assessment	Analytical Category	Definition/Nutrient Content Values	Consumption Categories	Adjusted OR (95% CI)	Adjusted Variables
Risch, 1985 [21], Canada	246 (35–79 years/by province-wide tumor registries, and surgical, pathology, and medical records)	246 population-based	1979–1982	diet frequent questionnaire	Nitrates mg/day	estimated by matching FFQ food items/food composition tables were modified and extended to Canadian items	NA	1.0 (Referent)	NA
								0.66 (0.54–0.81)	
					Nitrites mg/day			1.0 (Referent)	
								1.71 (1.24–2.37)	
Buiatti, 1990 [22], Italy	1016 (≤75 years/histologic confirmation)	1159 population-based	1985–1987	146-item questionnaire	Nitrates mg/day	estimated by matching questionnaire food items/using several Italian sources	53	1.0 (Referent)	Non-dietary variables and kilocalories
							81	0.90 (0.70–1.10)	
							103	0.90 (0.60–1.10)	
							130	0.70 (0.50–0.90)	
							193	0.90 (0.70–1.20)	
					Nitrites mg/day		2.1	1.0 (Referent)	
							2.8	1.00 (0.80–1.40)	
							3.4	1.20 (0.90–1.70)	
							4.1	1.40 (1.00–2.00)	
							5.9	1.90 (1.30–2.70)	
Boeing, 1991 [23], Germany	143 (32–80 years/histologically confirmed)	579 hospital-based	1985–1988	74-item standardized questionnaire	Nitrates Quintiles	estimated by matching questionnaire food items/German Federal Agency of Nutrition	Q1	1.0 (Referent)	Age, sex, and hospital
							Q2	0.93 (0.53–1.64)	
							Q3	0.61 (0.32–1.19)	
							Q4	0.61 (0.30–1.27)	
							Q5	1.26 (0.59–2.70)	
Hansson, 1994 [24], Sweden	338 (40–79 years/histologically confirmed)	679 population-based	1989–1992	45-item FFQ	Nitrates mg/day	estimated by matching FFQ food items (considered loss in cooked dishes)/based on data from several Swedish sources	23	1.0 (Referent)	Age, gender, ascorbic acid, β-carotene. and α-tocopherol
							34	0.85 (0.57–1.25)	
							45	0.99 (0.65–1.52)	
							69	0.97 (0.60–1.59)	
La Vecchia, 1994 [25], Italy	723 (19–74 years/histologically confirmed)	2024 hospital-based	1985–1992	29-item standard questionnaire	Nitrates mg/die	estimated by matching questionnaire food items/based on Italian tables of food composition	62.95	1.0 (Referent)	Age, sex, education, family history of gastric cancer, body mass index, and total energy intake
							80.70	0.64 (0.49–0.83)	
							96.33	0.50 (0.38–0.67)	
							116.88	0.52 (0.39–0.70)	
							>116.88	0.43 (0.32–0.59)	
					Nitrites mg/die		1.91	1.0 (Referent)	
							2.41	0.98 (0.72–1.33)	
							2.94	0.99 (0.72–1.36)	
							3.64	1.15 (0.84–1.59)	
							>3.64	1.35 (0.96–1.88)	
Pobel, 1995 [26], France	92 (mean: 66.6 years, SD: 10.4/histologically confirmed)	128 hospital-based	1985–1988	diet history questionnaire	Nitrates Tertiles	derived from dairy products, meat and eggs, fish, flour products, fruit, vegetables, beverages/using a composition table based on literature data	T1	1.0 (Referent)	Age, sex, occupation and total calorie intake
							T2	0.49 (0.24–1.01)	
							T3	0.76 (0.38–1.50)	
					Nitrites Tertiles		T1	1.0 (Referent)	
							T2	0.83 (0.41–1.67)	
							T3	0.88 (0.44–1.79)	
					NDMA Tertiles		T1	1.0 (Referent)	
							T2	4.13 (0.93–18.27)	
							T3	7.00 (1.85–26.46)	
La Vecchia, 1995 [27], Italy	746 (19–74 years/histologically confirmed)	2053 hospital-based	1985–1993	29-item structured questionnaire	NDMA μg/day	estimated by matching questionnaire food items/based Italian survey on selected foods or from other published data	≤0.13	1.0 (Referent)	Age, sex, education, family history of gastric cancer, combined food score index, intake of β-carotene, vitamin C, total calories, nitrite and nitrate intake
							0.13–0.19	1.11 (0.90–1.40)	
							>0.19	1.37 (1.10–1.70)	
De Stefani, 1998 [28], Uruguay	340 (25–84 years/microscopically confirmed)	698 hospital-based	1993–1996	FFQ	NDMA μg/day	derived from fried, broiled, or salted meat/according to previous literature data	≤0.14	1.0 (Referent)	Age, sex, residence, urban/rural status, tobacco duration, total alcohol consumption
							0.15–0.18	2.07 (1.36–3.18)	
							0.19–0.26	3.23 (2.13–4.89)	
							≥0.27	3.62 (2.38–5.51)	
Palli, 2001 [29], Italy	382 (<50 years, 30 cases; 50–64 years, 130 cases; > 64 years, 222 cases/histologically confirmed)	561 population-based	1985–1987	181-item FFQ	Nitrates mg/day	estimated by matching FFQ food items/based on Italian food composition tables estimated by matching FFQ food items/based on Italian food composition tables	62.6	1.0 (Referent)	Age, sex, social class, family history of gastric cancer, area of rural residence, BMI, total energy and the residuals of each nutrient of interest.
							93.2	0.70 (0.50–1.00)	
							132.9	0.60 (0.40–0.90)	
					Nitrites mg/day		2.5	1.0 (Referent)	
							3.5	1.40 (1.00–2.00)	
							5.4	1.40 (1.00–2.00)	
					NDMA		0.12	1.0 (Referent)	
							0.20	1.10 (0.80–1.60)	
							0.33	1.10 (0.80–1.50)	
Engel, 2003 [30], USA	629 (30–79 years/histologic reports from surgery, radiology, and endoscopy)	695 population-based	1993–1995	FFQ	Nitrites mg/day	estimated by matching FFQ food items/based on a nitrite database used in North America	Men, Women 1.7–5.8, 1.9–5.3 5.9–7.5, 5.4–6.9 7.6–9.9, 7.0–9.1 10–39.2, 9.2–31.2	1.0 (Referent)	NA
								1.50 (1.00–2.40)	
								1.80 (1.10–3.00)	
								2.50 (1.40–4.30)	
López-Carrillo, 2004 [31], Mexico	211 (≥20 years/histologically confirmed)	454 hospital-based	1994–1996	semi-quantitative questionnaire	Nitrites portions/day	derived from specific food consumption that is typical of each geographical region	0–0.11	1.0 (Referent)	Age, gender, residence, energy change in socioeconomic level, years of education, Hp/CagA status, and ascorbic acid
							0.12–0.26	0.95 (0.62–1.46)	
							0.27–2.25	1.24 (0.81–1.90)	
Kim, 2007 [32], Korea	136 (mean: 57.2 years, SD: 13.9/histologically confirmed)	136 hospital-based	1997–1998	84-item semiquantitative FFQ	Nitrates mg/day	estimated by matching FFQ food items/base on National Nutrition Survey Report in Koera	240	1.0 (Referent)	Age, sex, socioeconomic status, family history, refrigerator use, H. pylori infection, and foods
							458	1.13 (0.54–2.36)	
							811	1.13 (0.42–3.06)	
Ward, 2008 [33], USA	79 (≥21 years/histologically confirmed)	321 population-based	1988–1994	short Health Habits and History Questionnaire	Nitrates mg/day	derived from vegetables, processed meats, and water/based on previous published literature	<16.9	1.0 (Referent)	Year of birth, gender, education, smoking, alcohol, total calories, vitamin C, fiber, and carbohydrate
							16.9–26.2	1.20 (0.60–2.50)	
							26.2–38.8	1.40 (0.70–2.90)	
							>38.8	1.60 (0.70–3.60)	
					Nitrites mg/day	derived from breads, cereals, processed meats/based on previous published literature	<0.36	1.0 (Referent)	
							0.36–0.52	1.10 (0.40–2.70)	
							0.52–0.67	0.80 (0.30–2.20)	
							>0.67	1.10 (0.30–3.40)	
Hernández-Ramírez, 2009 [34], Mexico	228 (median: 59 years, P25-P75: 49–67 years/histologically confirmed)	467 population-based	2004–2005	127-item FFQ	Nitrates mg/day	estimated by matching FFQ food items/based on several published literature	≤90.4	1.0 (Referent)	Energy, age, gender, Hp/CagA status, schooling and consumptions of salt, chili, and alcohol
							>90.4–141.7	0.93 (0.62–1.39)	
							>141.7	0.61 (0.39–0.96)	
					Nitrites mg/day		≤1.0	1.0 (Referent)	
							>1.0–1.2	1.07 (0.69–1.65)	
							>1.2	1.52 (0.99–2.34)	
Navarro Silvera, 2011 [35], USA	255 cardia, 352 non-cardia (30–79 years/pathology reports)	687 population-based	1993–1995	104-item FFQ	Nitrites Quartiles	estimated by matching FFQ food items/based on Nutrition Coding Center Nutrient Data system	Q1 Q2 Q3 Q4	Cardia	Gender, age, site, race, income, education, proxy status, and energy intake
								1.0 (Referent)	
								1.13 (0.70–1.82)	
								1.75 (1.03–2.96)	
								1.82 (0.91–3.65)	
								Non-Cardia	
								1.0 (Referent)	
								1.89 (1.23–2.92)	
								2.03 (1.23–3.35)	
								2.40 (1.25–4.62)	

RR: relative risk; CI: confidence interval; BMI: body mass index; FFQ: food frequency questionnaire; SD: standard error; NA: Not Applicable.

### 3.2. Dietary Nitrates, Nitrites, and NDMA Intake and the Risk of Gastric Cancer

**Figure 2:**
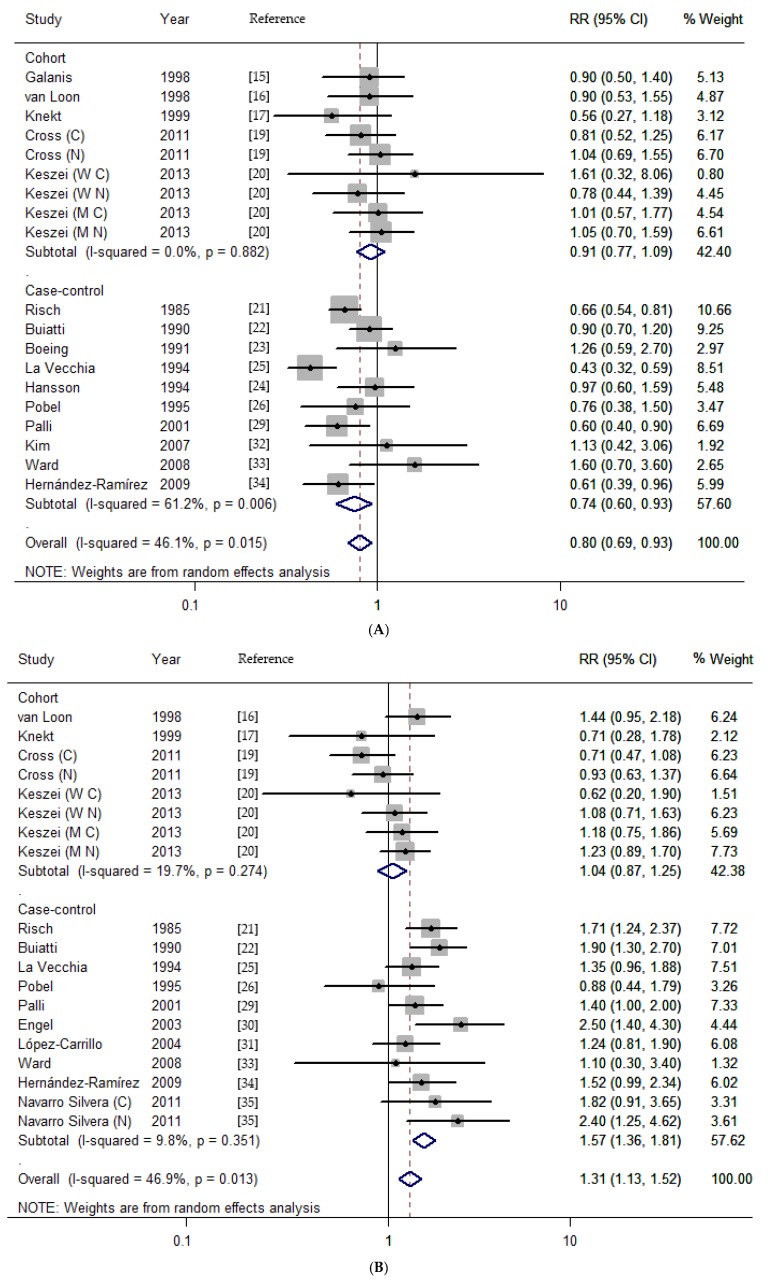

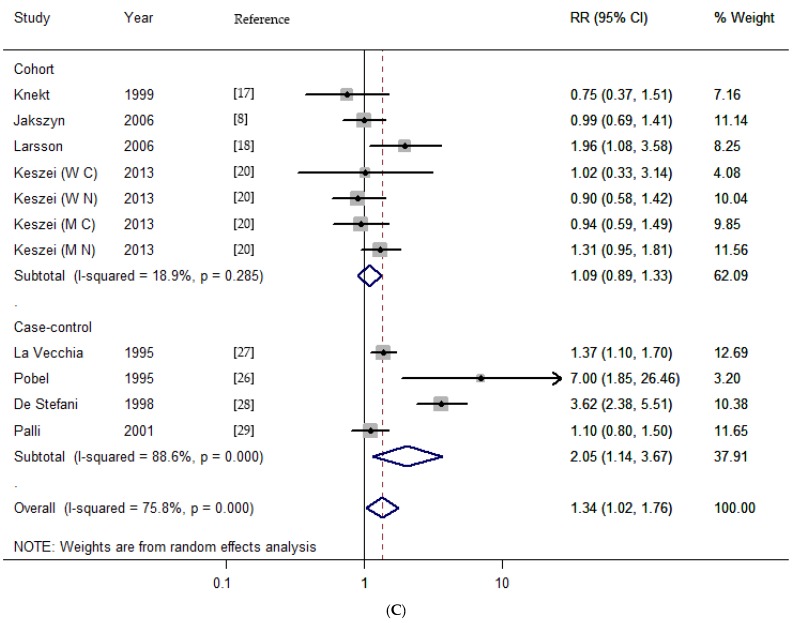
Dietary nitrates, nitrites and NDMA intake and the risk of gastric cancer for the highest *versus* lowest categories. (**A**) nitrates; (**B**) nitrites; (**C**) NDMA. (C, cardia; N, non-cardia; M, male; W, women).

There were nine cohort studies and 10 case-control studies of the relationship between the nitrates intake and gastric cancer risk [15,16,17,19,20,21,22,23,24,25,26,29,32,33,34]. Comparing the highest *versus* the lowest categories, the summary RR was 0.80 (95% CI, 0.69–0.93; Figure 2A) with significant heterogeneity (*I*^2^ = 46.1%, *p* = 0.015). To explore the heterogeneity, we conducted subgroup analysis according to some key characteristics. In stratified analysis by study design, significant inverse association was observed in population-based case-control studies (RR, 0.76; 95% CI, 0.62–0.94) with acceptable heterogeneity (*I*^2^ = 47.8%, *p* = 0.088). Stratifying by geographic area, the RR was 0.79 (95% CI, 0.64–0.98) in Europe. Besides, the associations between nitrates intake and risk of stomach cancer were similar in these subgroups (publication year < 2000, sample size < 2000, quality score < 7 stars; Table 3).

In pooled analysis of eight cohort studies and 10 case-control studies for nitrites [16,17,19,20,21,22,25,26,29,30,31,33,34,35], a significant association was observed. Overall, individuals with highest nitrites consumption, compared with the lowest, increased the risk of gastric cancer (pooled RR, 1.31; 95% CI, 1.13–1.52, Figure 2B). In the subgroup analysis by study design, the association was detected in both population-based case-control studies (RR, 1.72; 95% CI, 1.47–2.02) and hospital-based case-control studies (RR, 1.25; 95% CI, 1.09–1.44) with no heterogeneity. The risk for developing gastric cancer was significantly higher in Europe (RR: 1.30; 95% CI, 1.12–1.50). The risk effect of nitrites was also found in subgroups (publication year, before and after 2000; sample size < 2000; quality score < 7 stars; Table 3).

A total seven cohort studies and four case-control studies were pooled together to assess the association between NDMA consumption and stomach cancer risk [17,18,20,26,27,28,29,36]. The pooled RR for high *versus* low intake was 1.34 (95% CI, 1.02–1.76), with obvious evidence of heterogeneity (*I*^2^ = 75.8%, *p* < 0.001; Figure 2C). Study design, geographic area, cancer type, publication years, and sample size in association of NDMA consumption and gastric cancer were assessed separately. These RR estimates obtained from these subgroups showed no significant association (Table 3). Additionally, a slight association was observed in high quality studies (score ≥ 7 stars; RR, 1.30; 95% CI, 0.97–1.75).

**Table 3:** Stratified analysis of the association between nitrates, nitrites, and NDMA intake and stomach cancer risk.

Variable	Nitrates					Nitrites					NDMA				
	*n* ^a^	RR (95% CI)	Heterogeneity Test			*n* ^a^	RR (95% CI)	Heterogeneity Test			*n* ^a^	RR (95% CI)	Heterogeneity Test		
			Q	*p* ^b^	*I^2^*%			Q	*p* ^b^	*I^2^*%			Q	*p* ^b^	*I^2^*%
Total	19	0.80 (0.69–0.93)	31.39	0.015	46.1	19	1.31 (1.13–1.52)	33.87	0.013	46.9	11	1.34 (1.02–1.76)	41.35	<0.001	75.8
Study design															
Cohort	9	0.91 (0.77–1.09)	3.71	0.882	0.0	8	1.04 (0.87–1.25)	8.71	0.274	19.7	7	1.09 (0.89–1.33)	7.4	0.258	18.9
Case-control															
Population based	6	0.76 (0.62–0.94)	9.58	0.088	47.8	8	1.72 (1.47–2.02)	5.21	0.634	0.0	1	1.10 (0.80–1.50)	NA	NA	NA
Hospital based	4	0.75 (0.42–1.35)	9.91	0.019	69.7	3	1.25 (1.09–1.44)	1.16	0.559	0.0	3	2.81 (1.16–6.80)	20.54	<0.001	90.3
Geographic area															
Europe	12	0.79 (0.64–0.98)	24.03	0.013	54.2	10	1.30 (1.12–1.50)	10.14	0.339	11.3	10	1.18 (0.97–1.43)	16.89	0.050	46.7
North America	5	0.80 (0.62–1.04)	8.22	0.084	51.3	9	1.41 (1.06–1.87)	23.62	0.003	66.1	0	NA	NA	NA	NA
Other	2	0.94 (0.60–1.49)	0.16	0.690	0.0	0	NA	NA	NA	NA	1	3.62 (2.38–5.51)	NA	NA	NA
Cancer type															
cardia	3	0.90 (0.64–1.27)	0.88	0.644	0.0	4	1.01 (0.65–1.58)	6.64	0.084	54.8	3	0.87 (0.60–1.25)	0.66	0.718	0.0
non-cardia	3	0.99 (0.76–1.28)	0.79	0.672	0.0	4	1.22 (0.90–1.65)	6.21	0.102	51.7	3	1.14 (0.90–1.44)	1.81	0.404	0.0
Publication year															
<2000	9	0.75 (0.60–0.93)	19.56	0.012	59.1	6	1.46 (1.17–1.81)	7.38	0.194	32.3	4	2.02 (0.96–4.24)	25.62	<0.001	88.3
≥2000	10	0.86 (0.72–1.03)	10.59	0.305	15.0	13	1.26 (1.05–1.53)	23.40	0.025	48.7	7	1.12 (0.95–1.31)	6.22	0.399	3.6
Sample size															
<2000	8	0.76 (0.62–0.94)	9.99	0.189	29.9	9	1.56 (1.31–1.87)	9.26	0.321	13.6	3	2.69 (0.95–7.60)	24.06	<0.001	91.7
≥2000	11	0.82 (0.66–1.01)	22.50	0.013	55.6	10	1.15 (0.95–1.40)	17.85	0.037	49.6	8	1.16 (0.97–1.39)	9.84	0.198	28.9
Quality score															
<7 stars	7	0.70 (0.54–0.90)	16.83	0.010	64.3	6	1.58 (1.11–1.49)	7.76	0.170	35.6	2	2.47 (0.41–14.91)	7.04	0.008	85.8
≥7 stars	12	0.90 (0.77–1.04)	8.59	0.660	0.0	13	1.18 (0.99–1.40)	18.12	0.112	33.8	9	1.30 (0.97–1.75)	34.01	<0.001	76.5

RR: relative risk; CI: confidence interval; NA: Not Applicable. ^a^ Number of comparisons; ^b^*p* Value of Q-test for heterogeneity test.

### 3.3. Dose-Response Analysis

**Figure 3:**
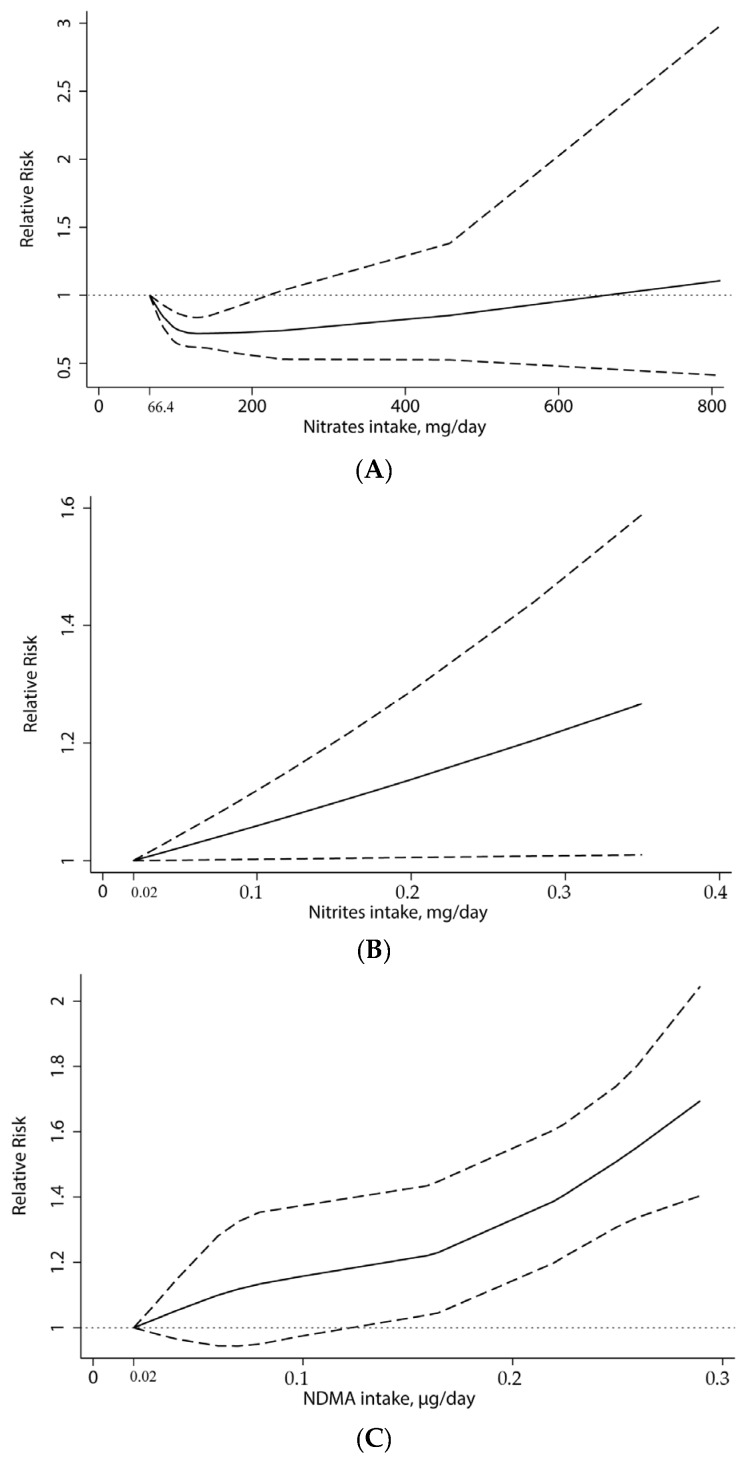
Dose-response analysis of dietary nitrates, nitrites and NDMA intake and the risk of gastric cancer. (**A**) the median value of the lowest reference interval (66.4 mg/day) was used to estimate all relative risks for nitrates; (**B**) the median value of the lowest reference interval (0.02 mg/day) was used to estimate all relative risks for nitrites; (**C**) the minimum value of the lowest reference interval (0.02 μg/day) was used to estimate all relative risks for NDMA. The solid line represents estimated RRs and dashed lines are their 95% CIs. The dotted line represents the null hypothesis of no association.

Four articles (7 studies) were eligible for the dose-response analysis of dietary nitrates intake and gastric cancer risk [16,20,25,32]. A nonlinear association was detected (*p*_non-linearity_ = 0.001, Figure 3A), with a significantly decreased risk at the nitrates intake level ranged from about 66.4 to 220 mg/day. After evaluating the dose-response pattern for nitrites (2 articles/5 studies) [16,20], some evidence of a linear association of gastric cancer was found (*p*_linearity_ = 0.041, Figure 3B). Accordingly, the summary RR for 0.1 mg/day increment of nitrites consumption was 1.07 (95% CI, 1.00–1.15) without heterogeneity (*p* = 0.876). Four papers (seven studies) were included in the dose-response analysis for NDMA [18,20,27,28]. We observed a nonlinear trend toward gastric cancer risk with increasing NDMA intake (*p*_non-linearity_ < 0.001), following an increase in the risk of NDMA intake up to 0.12 μg/day (Figure 3C).

### 3.4. Meta-Regression

As shown in Table 4, study design seemed to influence the overall heterogeneity mostly for the association of nitrates intake and gastric cancer risk. In univariate meta-regression analysis, study design alone could explain 58.14% (0.025/0.043) of the estimated between-study variance (τ^2^). When all the variables (study design, geographic area, and publication year) in the meta-regression model, the τ^2^ was reduced from 0.041 to 0.009 for nitrites, and from 0.139 to 0.026 for NDMA. Moreover, we found that study type was the main source of heterogeneity for nitrites, which interpreted 89.1% (0.041/0.046) of the τ^2^. Although geographic area could explain 92.1% (0.128/0.139) of the τ^2^ for NDMA, the subgroups stratifying by this variable still had non-negligible heterogeneity.

**Table 4:** Meta-regression analysis.

Variable	Nitrates			Nitrites			NDMA		
	Coefficient	*p* Value	95% CI	Coefficient	*p* Value	95% CI	Coefficient	*p* Value	95% CI
Study design	−0.154	0.184	−0.390 to 0.082	0.406	0.011	0.106 to 0.705	0.200	0.363	−0.286 to 0.686
Geographic area	0.023	0.846	−0.225 to 0.271	−0.030	0.831	−0.326 to 0.265	0.912	0.057	−0.035 to 1.860
Publication year	0.063	0.696	−0.275 to 0.400	−0.029	0.845	−0.343 to 0.285	0.097	0.807	−0.806 to 0.999

CI: confidence interval.

### 3.5. Sensitivity Analysis

We confirmed the associations between dietary nitrates, nitrites, and NDMA intake and gastric cancer risk were relatively stable using sensitivity analysis. After removing one study at a time, the ranges of pooled RRs were 0.67–0.97, 1.10–1.57, and 0.97–1.89 for nitrates, nitrites, and NDMA, respectively (Supplementary Figure S1). As shown in Supplementary Figure S1A, the study conducted by La Vecchia *et al.* seemed to cause the heterogeneity [25]. This phenomenon was verified through the Galbraith plot (Supplementary Figure S2). Exclusion of this study, the heterogeneity was not detected (*I*^2^ = 8.8%, *p* = 0.349), and the summary RRs were 0.82 (95% CI, 0.73–0.92) in the overall study and 0.79 (95% CI, 0.66–0.95) in the case-control study. Supplementary Figure S1C displayed that one study performed by De Stefani *et al.* influenced the overall pooled estimates for the association between NDMA intake and gastric cancer [28]. After this study was removed, the overall RR was 1.18 (95% CI, 0.97–1.43), with moderate heterogeneity (*I*^2^ = 46.7%, *p* = 0.050).

### 3.6. Publication Bias

**Figure 4:**
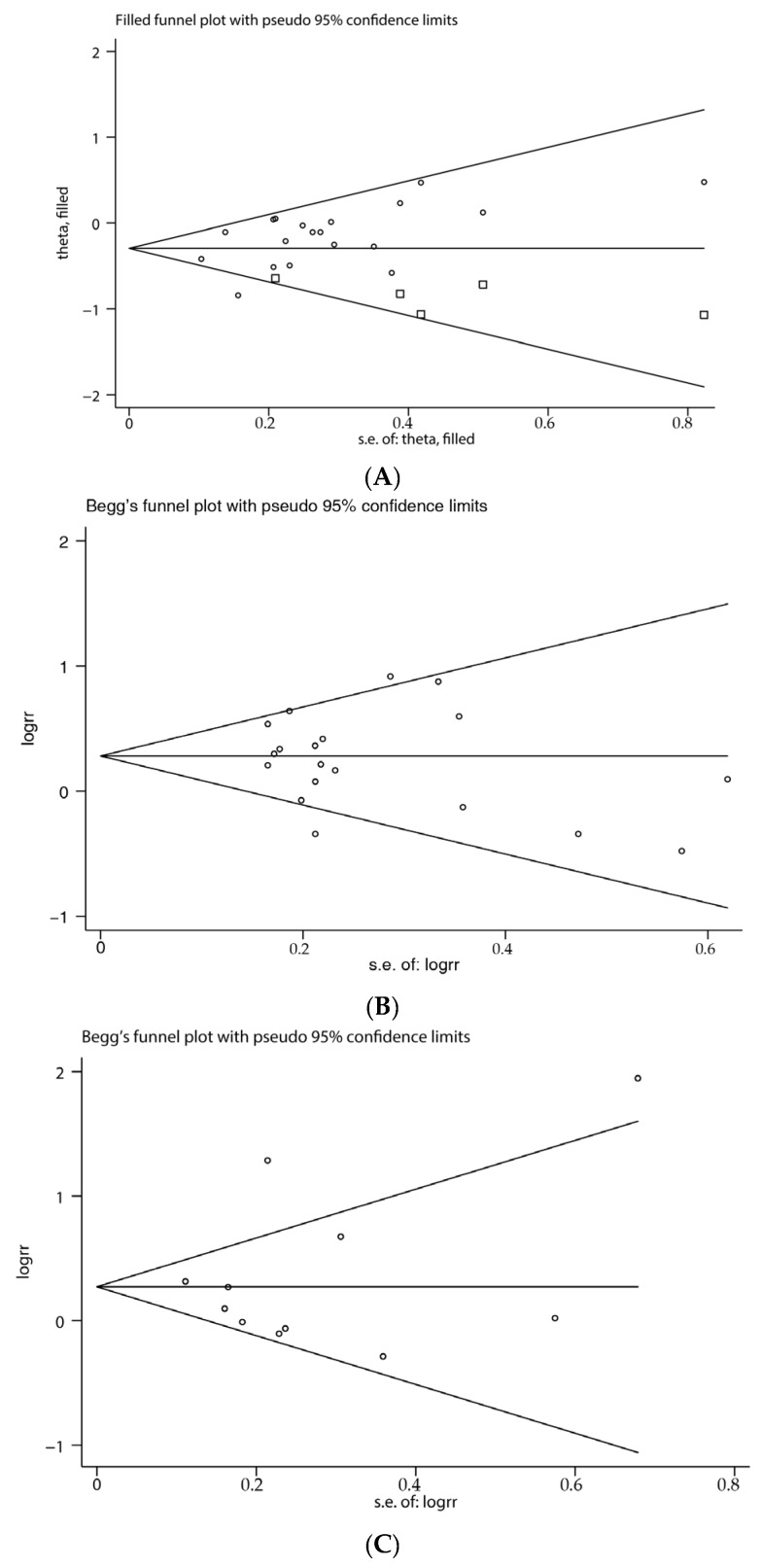
Funnel plot of nitrates, nitrites and NDMA consumption and gastric cancer risk. (**A**) Base on trim and fill method, hypothetical dummy studies indicated by squares are added to the genuine studies for nitrates; (**B**) nitrites; (**C**) NDMA.

As shown in Figure 4, these funnel plots did not reveal obvious signs of asymmetry. Moreover, the Egger and Begg test provided statistical evidence of bias for nitrates (Egger, *p* = 0.047; Begg, *p* = 0.327), nitrites (Egger, *p* = 0.542; Begg, *p* = 0.576), and NDMA (Egger, *p* = 0.821; Begg, *p* = 1.000). Adjusting the possible publication bias for nitrates using “trim and fill” method did not influence the conclusion (RR: 0.745; 95% CI, 0.646–0.860; Figure 4A).

## 4. Discussion

This is the first meat-analysis evaluating relationships between dietary nitrates, nitrites, and nitrosamines intake and the risk of gastric cancer. We found that consumption of food rich in nitrates was related to a decreased risk of gastric cancer, and that high intake of nitrites and NDMA resulted in an elevated risk of cancer. Stratifying analysis for study design, similar results were observed in the case-control studies, and the cohort studies also indicated the consequences of this trend. The dose-response analysis further showed that the inverse association between nitrates and stomach cancer appeared to be pronounced with nitrates intake level ranged from about 66.4 to 220 mg/day. Estimated in linear dose-response model for nitrites, the risk increased in gastric cancer was 7% corresponded to each 0.1 mg/day increment of nitrites intake. When daily NDMA intake reached 0.12 μg, the harmful effect to human became more obvious.

In order to understand the mechanisms of nitrates, nitrites, and NDMA, we need to know its chemical and potential biologic property. Nitrates and nitrites are two types of inorganic compounds, which compose of a single nitrogen atom (N) and a number of oxygen atoms (O); and the chemical symbols are NO_3_ and NO_2_ for nitrate and nitrite, respectively. It is believed that nitrates themselves are relatively inert, until they are reduced to nitrites. Nitrates can turn into nitrites by bacteria in the mouth and then be swallowed. As nitrites hit the highly acidic juices in stomach, it is converted to nitrous acid, which reacts with amines to form nitrosamines [37]. In our life, processed products such as meats are heated at high temperatures, the nitrites of which can also turn into nitrosamines. Animal models were used to test the carcinogenic potential of these chemical substances. In 2010, International Agency for Research on Cancer (IARC) have concluded that there was no substantial evidence implicating nitrates as animal carcinogen [38]. Besides, nitrites in combination with amines or amides were proved to be carcinogenic to animals. Most nitrosamines can induce animal carcinogenesis by causing gene mutation and DNA adductions. Thereafter, a well-done systematic review performed by Bryan *et al.* elaborated the animal toxicology of these molecules and drew a consistent conclusion [39]. Whereas human diet is a potentially modifiable exposure, it remains difficult to attribute the etiology of cancer to a single nutrient. Recently, the data from prospective cohort studies, indicating that estimated intake of nitrates, nitrites, and NDMA in the diet was not significantly associated with a risk of gastric cancer [15,16,17,19,20,40]. Thus, it is necessary to conduct a meta-analysis to reveal a trend that may not be obvious in a single study.

In the present study, high nitrates consumption demonstrated a protective effect for gastric cancer, in line with some previous studies [25,29,34]. Because dietary nitrates are mainly provided by vegetables, and its protection is likely to be reflected by fiber, vitamin C, and other anti-oxidants. As is known to all, the daily intake of nitrates in Korea is highest, due to the consumption of nitrate-rich green leafy vegetables such as Kimchi, and this country is also a high-risk region for gastric cancer in Asia. Kim and his colleagues reported that the estimated values of nitrates from the Korea Food Balance Sheet (390–742 mg/day) were considerably higher, compared to European countries (52–156 mg/day) and China (422.8 mg/day). Their research also showed that a higher intake of nitrates was not related to a greater cancer risk [32]. In contrast, higher nitrates intake relative to anti-oxidants was associated with an increase the gastric cancer risk. Considering the collinearity of nitrates and antioxidant vitamins intake, we further took a meta-analysis of these studies that had adjusted for vitamin C, vegetables, or fruits, and the pooled RR was 0.97 (95% CI, 0.81–1.17) with no heterogeneity (*I*^2^ = 0.0%, *p* = 0.849) [16,19,20,33]. Therefore, studies with validated methodologies quantifying the source of exposure as much as possible in the diet are needed to validate this finding.

Strengths of our meta-analysis included the large number of total subjects (650,826 for nitrates; 663,634 for nitrites; 742,038 for NDMA), dose-response relationship, reliable sources of heterogeneity, and the stable results in the sensitivity analysis. Here, some limitations were pointed out as follows. First, food frequency questionnaires were used to record the usual dietary consumption and classify them to estimate daily nitrates, nitrites, and NDMA intake. As a result, measurement error in different studies was inevitable, which might contribute to attenuation of the true relationship [41]. Second, there was a wide range of nitrates/nitrites/NDMA intake values between the lowest and highest categories, which might lead to the heterogeneity in the pooled analysis and conclusions limited. Third, only few articles were available for the stratified analysis of cancer type (cardia and non-cardia gastric cancer), and the dose-response analysis, especially for the nitrites (two papers/five studies), so we should treat the results with caution. More well-designed studies with detailed clinical characteristics are needed to answer these questions more completely. Fourth, significant heterogeneity was detected for NDMA, even after we confined to the stratified analysis, heterogeneity still existed in the subgroups. Fifth, *Helicobacter pylori* infection is a well-known risk factor for the development of distal gastric cancer. In this meta-analysis, only three case-control studies concerned this problem [31,32,34]. Lastly, during the long follow-up for cohort studies, the level of nitrates, nitrites, and nitrosamines in food have been marked changed due to the development of food processing technology. In addition, participants may have changed their diets and eating habits. Therefore, further prospective studies with complete questionnaires and updated diet information timely are warranted.

As diet is a very complex exposure variable, knowledge of beneficial factors and risk factors provide us an opportunity to improve heath and even prevent cancer. According to the report from WCRF/AICR [42], non-starchy vegetables as well as fruits with a relatively high content of anti-oxidants, ascorbic acid, and fiber probably protect against stomach cancer. However, salt and also salt-preserved foods have been proposed for probably causing this cancer. There is limited evidence suggesting dietary nitrates, nitrites, and NDMA intake increase the cancer risk. A review of previous research, studies of low quality tended to support the hypothesis of an increased risk with consumption of nitrite intake, while most research including better designed and conducted studies regarded NDMA as a potential human carcinogen. These results were in accord with our stratified analysis by sample size and quality score.

## 5. Conclusions

In summary, this meta-analysis suggested that dietary nitrates intake was associated with a reduced risk of gastric cancer, and high consumption of nitrites and NDMA could increase the risk. Considering the limitations and confounding factors, we could not absolutely confirm the reliability of these findings. More well-designed large prospective studies are needed to help us understand these substances in the etiology of gastric cancer.

## Supplementary Materials

The following are available online at www.mdpi.com/2072-6643/7/12/5505/s1, Table S1: Methodologic quality of cohort studies included in the meta-analysis, Table S2: Methodologic quality of case-control studies included in the meta-analysis, Figure S1: Influence analysis of the summary relative risks for dietary nitrates, nitrites and NDMA intake. (A) nitrates; (B) nitrites; (C) NDMA, Figure S2: Galbraith plots of nitrates intake and gastric cancer. The central solid line and two outer parallel lines represent the estimated RRs and 95% CIs, respectively.
