# Supplementary material for: Dietary Nitrates, Nitrites, and Nitrosamines Intake and the Risk of Gastric Cancer: A Meta-Analysis
Source: Nutrients. 2015 Dec 1;7(12):9872–95. doi: 10.3390/nu7125505 (PMC4690057; doi:10.3390/nu7125505)
Supplement: Supplementary file 1 [file nutrients-07-05505-s001.docx]

Supplementary Materials: Dietary Nitrates, Nitrites, and Nitrosamines Intake and the Risk of Gastric Cancer: A Meta-Analysis

Peng Song ^1,†^, Lei Wu ^2,†^ and Wenxian Guan ^1,^*

**Table S1.** Methodologic quality of cohort studies included in the meta-analysis.

| **First Author, Year** | **Representativeness of the Exposed Cohort** | **Selection of the Unexposed Cohort** | **Ascertainment of Exposure** | **Outcome of Interest Not Present at Start of Study** | **Control for Important Factor or Additional Factors ^a^** | **Assessment of Outcome** | **Follow-up Long Enough for Outcomes to Occur ^b^** | **Adequacy of Follow-up of Cohorts ^c^** | **Total Quality Scores** |
| --- | --- | --- | --- | --- | --- | --- | --- | --- | --- |
| Galanis, 1998 [1] | ☆ | ☆ | ☆ | ☆ | ☆ | ☆ | ☆ | ☆ | 8 |
| Van Loon, 1998 [2] | - | ☆ | ☆ | ☆ | ☆☆ | ☆ | ☆ | ☆ | 8 |
| Knekt, 1999 [3] | ☆ | ☆ | ☆ | ☆ | ☆ | ☆ | ☆ | - | 7 |
| Jakszyn, 2006 [4] | ☆ | ☆ | ☆ | ☆ | ☆☆ | ☆ | ☆ | - | 8 |
| Larsson, 2006 [5] | ☆ | ☆ | ☆ | ☆ | ☆ | ☆ | ☆ | - | 7 |
| Cross, 2011 [6] | ☆ | ☆ | ☆ | ☆ | ☆☆ | ☆ | ☆ | - | 8 |
| Keszei, 2013 [7] | ☆ | ☆ | ☆ | ☆ | ☆☆ | ☆ | ☆ | ☆ | 9 |

^a^ A maximum of 2 stars could be awarded for this item. Studies that controlled for age and sex received 1 star, and studies that controlled for other important confounders such as vegetable intake, fruit intake or vitamin C received an additional star; ^b^ A cohort study with a follow-up time longer than 5 years was assigned 1 star; ^c^ A cohort study with a follow-up rate greater than 80% was assigned 1 star.

**Table S2.** Methodologic quality of case-control studies included in the meta-analysis.

| **First Author, Year** | **Adequate Definition of Cases** | **Representativeness of Cases** | **Selection of Controls** | **Definition of Controls** | **Control for Important Factor or Additional Factors ^a^** | **Exposure Assessment** | **Same Method of Ascertainment for All Subjects** | **Nonresponse Rate ^b^** | **Total Quality Scores** |
| --- | --- | --- | --- | --- | --- | --- | --- | --- | --- |
| Risch, 1985 [8] | ☆ | ☆ | ☆ | ☆ | - | ☆ | ☆ | - | 6 |
| Buiatti, 1990 [9] | ☆ | ☆ | ☆ | - | - | ☆ | ☆ | ☆ | 6 |
| Boeing, 1991 [10] | ☆ | ☆ | - | ☆ | ☆ | ☆ | ☆ | - | 6 |
| Hansson, 1994 [11] | ☆ | ☆ | ☆ | ☆ | ☆ | ☆ | ☆ | ☆ | 8 |
| La Vecchia, 1994 [12] | ☆ | ☆ | - | ☆ | ☆ | ☆ | ☆ | - | 6 |
| Pobel, 1995 [13] | - | ☆ | ☆ | ☆ | ☆ | ☆ | ☆ | - | 6 |
| La Vecchia, 1995 [14] | ☆ | ☆ | - | ☆ | ☆☆ | ☆ | ☆ | - | 7 |
| De Stefani, 1998 [15] | ☆ | ☆ | - | ☆ | ☆ | ☆ | ☆ | ☆ | 7 |
| Palli, 2001 [16] | ☆ | ☆ | ☆ | - | ☆ | ☆ | ☆ | - | 6 |
| Engel, 2003 [17] | ☆ | ☆ | ☆ | ☆ | - | ☆ | ☆ | - | 6 |
| López-Carrillo, 2004 [18] | ☆ | ☆ | - | ☆ | ☆ | ☆ | ☆ | ☆ | 7 |
| Kim, 2007 [19] | ☆ | ☆ | - | ☆ | ☆ | ☆ | ☆ | - | 6 |
| Ward, 2008 [20] | ☆ | ☆ | ☆ | ☆ | ☆☆ | ☆ | ☆ | ☆ | 9 |
| Hernández-Ramírez, 2009 [21] | ☆ | ☆ | ☆ | ☆ | ☆ | ☆ | ☆ | ☆ | 8 |
| Navarro Silvera, 2011 [22] | ☆ | ☆ | ☆ | ☆ | ☆ | ☆ | ☆ | ☆ | 8 |

^a^ A maximum of 2 stars could be awarded for this item. Studies that controlled for age and sex received 1 star, and studies that controlled for other important confounders such as vegetable intake, fruit intake or vitamin C received an additional star; ^b^ One star was assigned if there was no significant difference in the response rate between case and control subjects by using the chi-square test (*p* > 0.05).


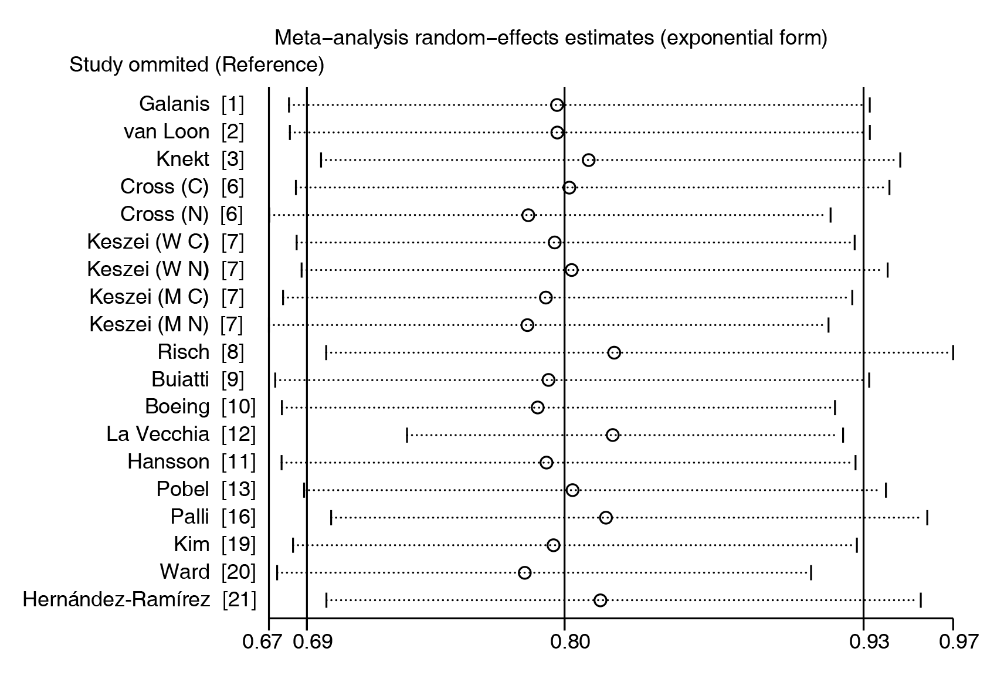


A


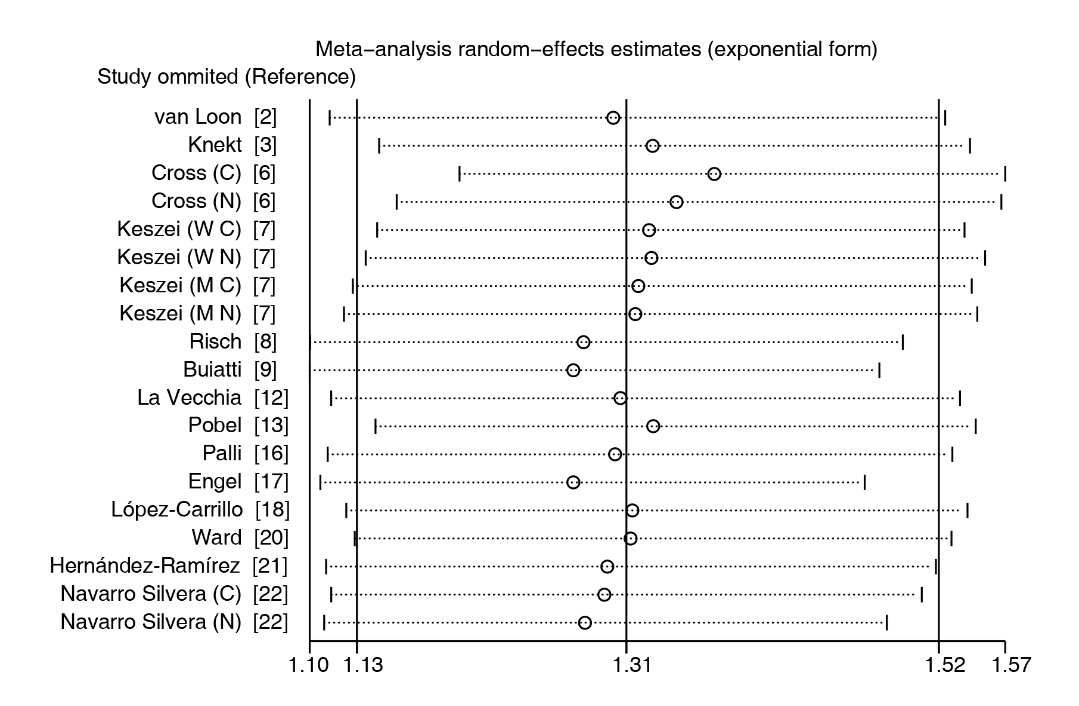


B


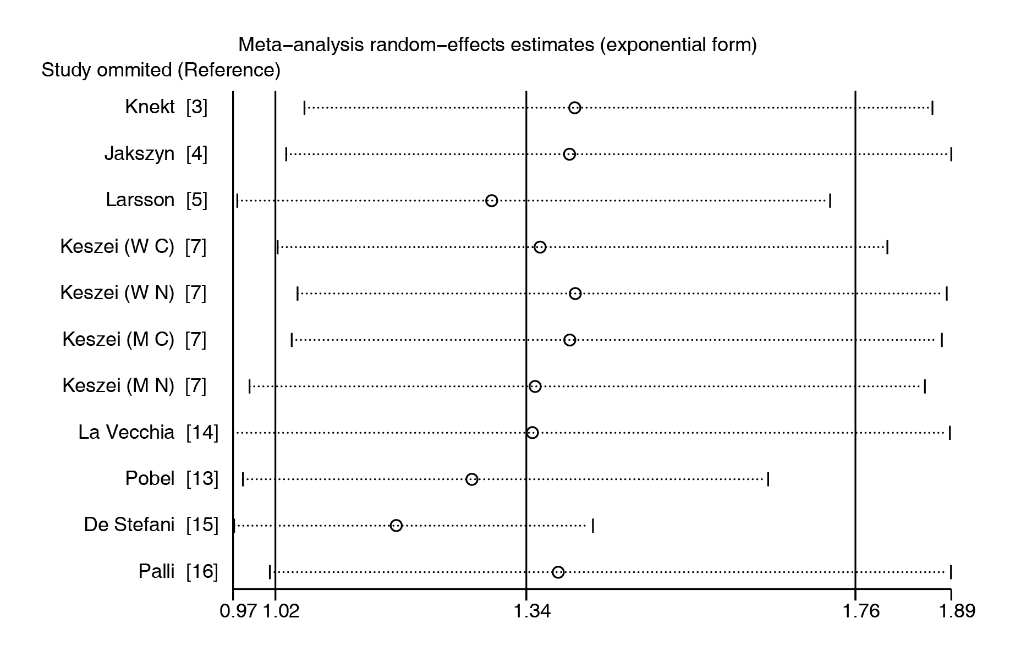


C

**Figure S1.** Influence analysis of the summary relative risks for dietary nitrates, nitrites and NDMA intake.
(**A**) nitrates; (**B**) nitrites; (**C**) NDMA.

**
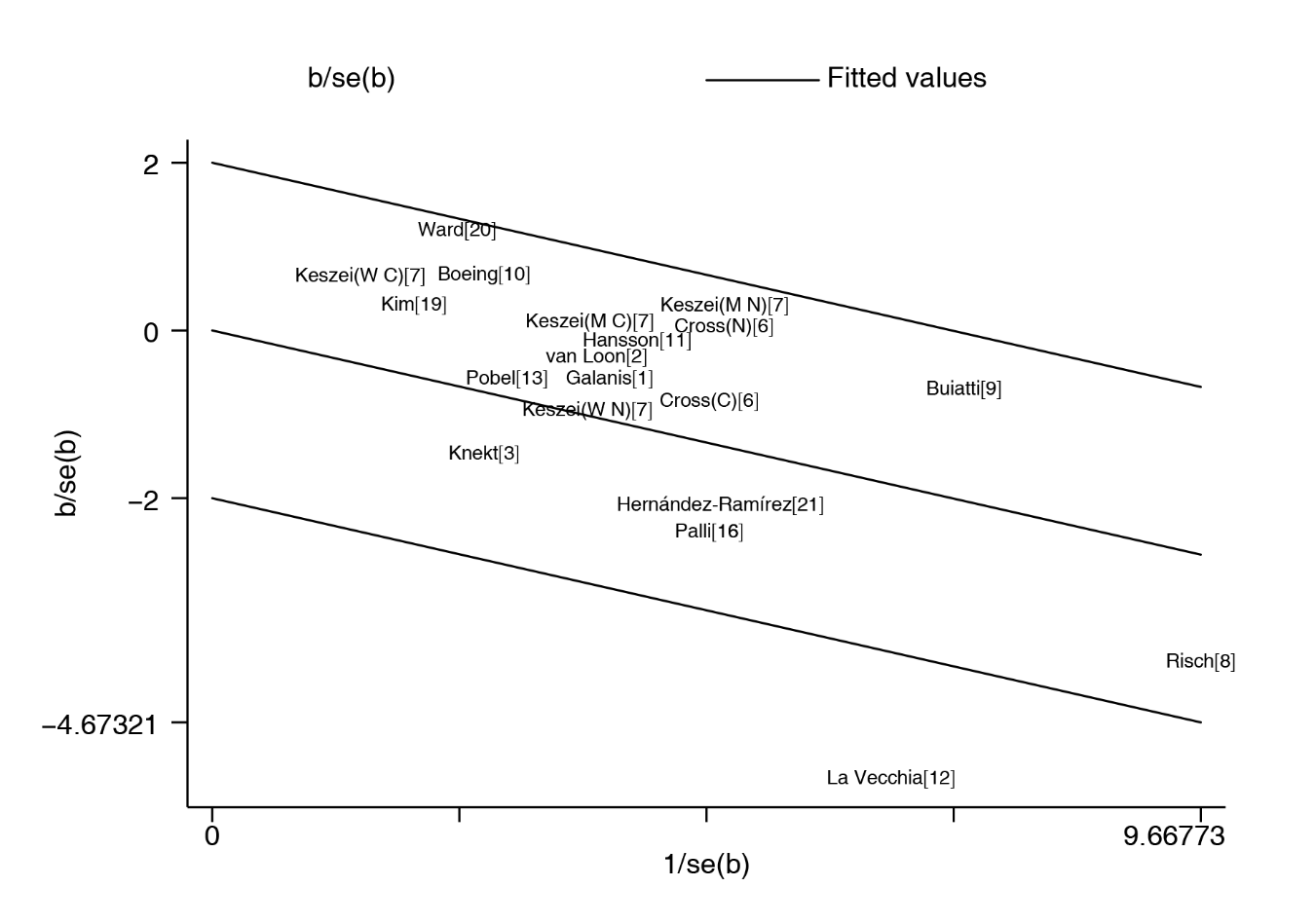
**

**Figure S2.** Galbraith plots of nitrates intake and gastric cancer. The central solid line and two outer parallel lines represent the estimated RRs and 95% CIs, respectively.

References

1. Galanis, D.J.; Kolonel, L.N.; Lee, J.; Nomura, A. Intakes of selected foods and beverages and the incidence of gastric cancer among the Japanese residents of Hawaii: A prospective study. *Int. J. Epidemiol.* **1998**, *27*, 173–180.
2. Van Loon, A.J.; Botterweck, A.A.; Goldbohm, R.A.; Brants, H.A.; van Klaveren, J.D.; van den Brandt, P.A. Intake of nitrate and nitrite and the risk of gastric cancer: A prospective cohort study. *Br. J. Cancer* **1998**, *78*, 129–135.
3. Knekt, P.; Jarvinen, R.; Dich, J.; Hakulinen, T. Risk of colorectal and other gastro-intestinal cancers after exposure to nitrate, nitrite and *N*-nitroso compounds: A follow-up study. *Int. J. Cancer* **1999**, *80*, 852–856.
4. Jakszyn, P.; Agudo, A.; Berenguer, A.; Ibanez, R.; Amiano, P.; Pera, G.; Ardanaz, E.; Barricarte, A.; Chirlaque, M.D.; Dorronsoro, M.; *et al.* Intake and food sources of nitrites and *N*-nitrosodimethylamine in spain. *Public Health Nutr.* **2006**, *9*, 785–791.
5. Larsson, S.C.; Bergkvist, L.; Wolk, A. Processed meat consumption, dietary nitrosamines and stomach cancer risk in a cohort of Swedish women. *Int. J. Cancer* **2006**, *119*, 915–919.
6. Cross, A.J.; Freedman, N.D.; Ren, J.; Ward, M.H.; Hollenbeck, A.R.; Schatzkin, A.; Sinha, R.; Abnet, C.C. Meat consumption and risk of esophageal and gastric cancer in a large prospective study. *Am. J. Gastroenterol.* **2011**, *106*, 432–442.
7. Keszei, A.P.; Goldbohm, R.A.; Schouten, L.J.; Jakszyn, P.; van den Brandt, P.A. Dietary *N*-nitroso compounds, endogenous nitrosation, and the risk of esophageal and gastric cancer subtypes in the Netherlands cohort study. *Am. J. Clin. Nutr.* **2013**, *97*, 135–146.
8. Risch, H.A.; Jain, M.; Choi, N.W.; Fodor, J.G.; Pfeiffer, C.J.; Howe, G.R.; Harrison, L.W.; Craib, K.J.; Miller, A.B. Dietary factors and the incidence of cancer of the stomach. *Am. J. Epidemiol.* **1985**, *122*, 947–959.
9. Buiatti, E.; Palli, D.; Decarli, A.; Amadori, D.; Avellini, C.; Bianchi, S.; Bonaguri, C.; Cipriani, F.; Cocco, P.; Giacosa, A.; *et al.* A case-control study of gastric cancer and diet in Italy: II. Association with nutrients. *Int. J. Cancer* **1990**, *45*, 896–901.
10. Boeing, H.; Frentzel-Beyme, R.; Berger, M.; Berndt, V.; Gores, W.; Korner, M.; Lohmeier, R.; Menarcher, A.; Mannl, H.F.; Meinhardt, M.; *et al.* Case-control study on stomach cancer in Germany. *Int. J. Cancer* **1991**, *47*, 858–864.
11. Hansson, L.E.; Nyren, O.; Bergstrom, R.; Wolk, A.; Lindgren, A.; Baron, J.; Adami, H.O. Nutrients and gastric cancer risk. A population-based case-control study in Sweden. *Int. J. Cancer* **1994**, *57*, 638–644.
12. La Vecchia, C.; Ferraroni, M.; D’Avanzo, B.; Decarli, A.; Franceschi, S. Selected micronutrient intake and the risk of gastric cancer. *Cancer Epidemiol. Biomarkers Prev.* **1994**, *3*, 393–398.
13. Pobel, D.; Riboli, E.; Cornee, J.; Hemon, B.; Guyader, M. Nitrosamine, nitrate and nitrite in relation to gastric cancer: A case-control study in Marseille, France. *Eur. J. Epidemiol.* **1995**, *11*, 67–73.
14. La Vecchia, C.; D’Avanzo, B.; Airoldi, L.; Braga, C.; Decarli, A. Nitrosamine intake and gastric cancer risk. *Eur. J. Cancer Prev.* **1995**, *4*, 469–474.
15. De Stefani, E.; Boffetta, P.; Mendilaharsu, M.; Carzoglio, J.; Deneo-Pellegrini, H. Dietary nitrosamines, heterocyclic amines, and risk of gastric cancer: A case-control study in Uruguay. *Nutr. Cancer* **1998**, *30*, 158–162.
16. Palli, D.; Russo, A.; Decarli, A. Dietary patterns, nutrient intake and gastric cancer in a high-risk area of Italy. *Cancer Causes Control* **2001**, *12*, 163–172.
17. Engel, L.S.; Chow, W.H.; Vaughan, T.L.; Gammon, M.D.; Risch, H.A.; Stanford, J.L.; Schoenberg, J.B.; Mayne, S.T.; Dubrow, R.; Rotterdam, H.; *et al.* Population attributable risks of esophageal and gastric cancers. *J. Natl. Cancer Inst.* **2003**, *95*, 1404–1413.
18. López-Carrillo, L.; Torres-López, J.; Galván-Portillo, M.; Muñoz, L.; López-Cervantes, M. Helicobacter pylori-caga seropositivity and nitrite and ascorbic acid food intake as predictors for gastric cancer. *Eur. J. Cancer* **2004**, *40*, 1752–1759.
19. Kim, H.J.; Lee, S.S.; Choi, B.Y.; Kim, M.K. Nitrate intake relative to antioxidant vitamin intake affects gastric cancer risk: A case-control study in Korea. *Nutr. Cancer* **2007**, *59*, 185–191.
20. Ward, M.H.; Heineman, E.F.; Markin, R.S.; Weisenburger, D.D. Adenocarcinoma of the stomach and esophagus and drinking water and dietary sources of nitrate and nitrite. *Int. J. Occup. Environ. Health* **2008**, *14*, 193–197.
21. Hernández-Ramírez, R.U.; Galván-Portillo, M.V.; Ward, M.H.; Agudo, A.; González, C.A.; Oñate-Ocaña, L.F.; Herrera-Goepfert, R.; Palma-Coca, O.; López-Carrillo, L. Dietary intake of polyphenols, nitrate and nitrite and gastric cancer risk in Mexico city. *Int. J. Cancer* **2009**, *125*, 1424–1430.
22. Silvera, S.A.N.; Mayne, S.T.; Risch, H.A.; Gammon, M.D.; Vaughan, T.; Chow, W.H.; Dubin, J.A.; Dubrow, R.; Schoenberg, J.; Stanford, J.L.; *et al.* Principal component analysis of dietary and lifestyle patterns in relation to risk of subtypes of esophageal and gastric cancer. *Ann. Epidemiol.* **2011**, *21*, 543–550.
